# Diversity dynamics of aerobic anoxygenic phototrophic bacteria in a freshwater lake

**DOI:** 10.1111/1758-2229.13131

**Published:** 2022-12-12

**Authors:** Cristian Villena‐Alemany, Izabela Mujakić, Petr Porcal, Michal Koblížek, Kasia Piwosz

**Affiliations:** ^1^ Laboratory of Anoxygenic Phototrophs Institute of Microbiology of the Czech Academy of Sciences Třeboň Czechia; ^2^ Department of Ecosystem Biology, Faculty of Science University of South Bohemia České Budějovice Czechia; ^3^ Department of Hydrochemistry and Ecosystem Modelling, Biology Centre of the Czech Academy of Sciences Institute of Hydrobiology České Budějovice Czechia; ^4^ Department of Fisheries Oceanography and Marine Ecology National Marine Fisheries Research Institute Gdynia Poland

## Abstract

Aerobic anoxygenic photoheterotrophic (AAP) bacteria represent a functional group of prokaryotic organisms that harvests light energy using bacteriochlorophyll‐containing photosynthetic reaction centers. They represent an active and rapidly growing component of freshwater bacterioplankton, with the highest numbers observed usually in summer. Species diversity of freshwater AAP bacteria has been studied before in lakes, but its seasonal dynamics remain unknown. In this report, we analysed temporal changes in the composition of the phototrophic community in an oligo‐mesotrophic freshwater lake using amplicon sequencing of the *puf*M marker gene. The AAP community was dominated by phototrophic Gammaproteobacteria and Alphaproteobacteria, with smaller contribution of phototrophic Chloroflexota and Gemmatimonadota. Phototrophic Eremiobacteriota or members of Myxococcota were not detected. Interestingly, some AAP taxa, such as *Limnohabitans*, *Rhodoferax,* Rhodobacterales or Rhizobiales, were permanently present over the sampling period, while others, such as Sphingomonadales, Rhodospirillales or Caulobacterales appeared only transiently. The environmental factors that best explain the seasonal changes in AAP community were temperature, concentrations of oxygen and dissolved organic matter.

## INTRODUCTION

Photoheterotrophic bacteria represent an important component of freshwater bacterioplankton. These organisms harvest light energy but, as they do not fix carbon, require organic carbon to build biomass. Two main groups of aquatic photoheterotrophic bacteria are rhodopsin‐containing bacteria and aerobic anoxygenic phototrophic (AAP) bacteria. Rhodopsins are membrane‐bound proteins able to use light energy to translocate protons across the membrane. The proton gradient is used for ATP production (Lanyi, 2004; Spudich & Luecke, 2002). In contrast, AAP bacteria harvest light energy using photosynthetic complexes containing bacteriochlorophyll‐*a* (BChl‐*a*) (Koblížek, 2015; Yurkov & Csotonyi, 2009). Due to the captured energy, AAPs reduce utilization of organic carbon for respiration and increase their biomass yield up to 100% (Hauruseu & Koblížek, 2012; Piwosz et al., 2018). When compared with rhodopsin‐containing bacteria, AAP bacteria are more effective in producing energy from light (Kirchman & Hanson, 2013).

AAP bacteria contribute 1–22% of all prokaryote abundance in the euphotic layer of freshwater lakes (Čuperová et al., 2013; Fauteux et al., 2015; Mašín et al., 2008, 2012; Ruiz‐González et al., 2013; Ruiz‐González et al., 2020). They have, on average, larger cell size and exhibit faster growth and mortality rates than heterotrophic bacteria (Cepáková et al., 2016; Garcia‐Chaves et al., 2016). Recently, it has been shown that upon infra‐red illumination AAP bacteria reduce total microbial respiration by 15% and increase microbial production by 6% (Piwosz et al., 2022). All these findings indicate the importance of AAPs in microbial food webs and the freshwater carbon cycle (Fauteux et al., 2015; Koblížek et al., 2005).

The most common representatives of AAP bacteria in freshwaters are members of the order Burkholderiales (Gammaproteobacteria), such as *Limnohabitans*, *Rhodoferax* and *Polynucleobacter* and members of orders Sphingomonadales and Rhodobacterales (Alphaproteobacteria), with a minor contribution of Chloroflexota and Gemmatimonadota (Caliz & Casamayor, 2014; Fecskeová et al., 2019; Kasalický et al., 2018; Martinez‐Garcia et al., 2012; Mujakić et al., 2021; Salka et al., 2011). Unfortunately, most of these studies provided only snap‐shot information on AAP community composition from a single or infrequent sampling of multiple lakes, even though seasonal time series have shown AAP bacteria undergo large changes in abundance, activity and growth rates (Cepáková et al., 2016; Čuperová et al., 2013; Kolářová et al., 2019). This has likely also a large impact on AAP community composition, which remain unexplored. Therefore, we decided to investigate how the diversity changes during the year.

Here, we applied amplicon sequencing of the 16S rRNA gene and *puf*M marker gene for AAP bacteria to study temporal changes in total bacterial and AAP bacteria community composition in the epi‐ and the hypolimnion in a shallow, meso‐oligotrophic freshwater lake Cep. We hypothesized that the diversity of AAP bacteria would show distinct temporal patterns in the epi‐ and the hypolimnion, as had been observed for all bacteria (Zemskaya et al., 2020). We also hypothesized that the AAP community would be affected by different environmental factors than the overall bacterial community.

## EXPERIMENTAL PROCEDURES

### Sampling

Samples were collected from April to September 2016 from oligotrophic Cep lake (48°92′49.24″ N, 14°88′68.11″ E) located in the Třeboň Basin Protected Landscape Area, Czechia. The lake was created in the second half of the 20th century as a result of sand mining and was filled with groundwater seeping from the nearby river Lužnice. Water samples were collected from five depths: 0.5, 2, 5, 7 and 9 m using a Ruttner Water Sampler (model 11.003KC Denmark AS). Water was transported to the laboratory in closed plastic containers, which were pre‐rinsed three times with the sampled water and stored in a cooled box. Temperature and oxygen profiles were taken with an EXO1 multi‐parameter probe (YSI Inc., Yellow Springs, OH).

### Bacteria and AAP bacteria microscopy counts

Samples of 50 ml were fixed with buffered, sterile‐filtered paraformaldehyde (Penta, Prague, Czechia) to a final concentration of 1%, and 0.5 ml was filtered onto white polycarbonate filters (pore size 0.2 μm, Nucleopore, Whatman, Maidstone, UK). Cells were stained with 4′,6‐diamidino‐2‐phenylindole (DAPI) at concentration of 1 mg L^−1^ (Coleman, 1980). Total and AAP bacterial abundances were determined using an epifluorescence Zeiss Axio Imager.D2 microscope equipped with Collibri LED module illumination system (Carl Zeiss, Jena, Germany). Ten microphotographs were taken for every sample under 325–370 nm excitation and 420–470 nm emission wavelengths for DAPI fluorescence (total bacteria), 450–490 nm excitation and 600–660 nm emission wavelengths for autofluorescence from Chl‐*a* (algae and cyanobacteria), and combined 325–370 nm, 450–490 nm, 545–565 nm and 615–635 nm excitation and 645–850 emission wavelengths for autofluorescence from BChl‐*a* (AAP bacteria). As some part of Chl‐*a* autofluorescence is also visible in the infrared spectrum, only the IR‐positive cells that did not show any autofluorescence from Chl‐*a* were counted as AAP bacteria (Cottrell et al., 2006).

### *Nutrients and chlorophyll‐*a

Samples were filtered through glass fibre filters with 0.4 μm nominal porosity (GF‐5, Macherey‐Nagel, Düren, Germany). Concentrations of soluble reactive phosphorus (SRP) were determined spectrophotometrically (Kopáček & Hejzlar, 1993; Murphy & Riley, 1962). Concentrations of nitrate and ammonium were measured according to Procházková (Procházková, 1959) and Kopáček and Procházková (Kopáček & Procházková, 1993). DOC and dissolved nitrogen (DN) were determined by catalytic thermal combustion at 720°C in combination with chemiluminescence detection by Shimadzu TOC‐L equipped with TNM‐L Total Nitrogen module (Shimadzu, Kyoto, Japan).

For Chl‐*a* measurements, phytoplankton was collected by filtration onto GF‐5 glass fibre filters (Macherey‐Nagel). The filters were dried of excess water by gently pressing in a paper towel, and flush frozen in liquid nitrogen. Pigments were extracted in acetone‐methanol (7:2, v:v) mixture and analysed by HPLC as described in (Piwosz et al., 2020).

### DNA isolation

Water (between 250 and 700 ml) was filtered through sterile 0.2 μm Nucleopore Track‐Etch Membrane filter units (Whatman, Maidstone, United Kingdom). Filters were put inside sterile cryogenic vials, flash‐frozen in liquid nitrogen and stored at −80°C. DNA extraction was done using PowerWater DNA Isolation Kit (MO BIO Laboratories Inc., Carlsbad, CA).

DNA samples were pooled in equimolar concentrations for the subsequent analysis. The epilimnion samples included water collected from 0.5 and 2 m, and the hypolimnion ones from the deeper layers (5–9 m). Such division was based on the temperature profiles.

### Bacterial community analysis

Amplicons of 16S rRNA gene were prepared using the primers set 341F‐785R (Klindworth et al., 2013). PCR was performed in 20 μl reaction, using Phusion High‐Fidelity PCR MasterMix (Thermo Scientific, USA). Reaction conditions were as follow: 98°C for 3 min, 25 cycles at 98°C for 10 s, 60°C for 20 s, 72°C for 20 s and a final extension at 72°C for 3 min. The reactions for each sample were done in triplicates, pooled and purified from the gel using the Wizzard SV Gel and PCR clean system (Promega), and quantified with Qubit dsDNA HS assay. Amplicons were sequenced on Illumina MiSeq (2 × 250 bp) platform at Genomic Service of the Universitat Pompeu Fabra (Barcelona, Spain).

Obtained reads were quality checked using FastQC v0.11.7 (Babraham Bioinformatics, Cambridge, UK). The primer sequences were trimmed using Cutadapt v1.16 (Martin, 2011) and further analysis was done in the R/Bioconductor environment using the DADA2 package (version 1.12.1) (Callahan et al., 2016). Low quality reads were filtered out and cut (dada2::filterAndTrim, truncLen = c(225, 225), maxN = 0, maxEE = c(2,2), truncQ = 2, rm.phix = TRUE, compress = TRUE). Sequences were merged (dada2::mergePairs()), and using removeBimeraDenovo function (method = “pooled”), chimeras were removed and singletons and doubletons were eliminated using Phyloseq (McMurdie & Holmes, 2013) (phyloseq::filter_taxa(ps, function(x) sum(x > 3) > (0.2*length(x)), TRUE)) resulting in 685 amplicon sequence variants (ASV) (Table S1). The final ASV table contained from 18,551 to 71,613 reads per sample (average ± SD: 42,945 ± 14,271). Taxonomic assignment was done in DADA2 (dada2:: assignTaxonomy()) using the SILVA r138.1 database released on 27 August 2020. Graphs were done using phyloseq (McMurdie & Holmes, 2013) and ggplot2 (Wickham, 2009) packages.

### AAP bacteria community analysis

The composition of AAP bacteria community was analysed using the *puf*M gene. Amplicons for *puf*M gene were prepared using *puf*M UniF and *puf*M UniR primers (Yutin et al., 2005). The hypolimnion sample from 15/06 could not be amplified and was excluded in further analysis. The PCR was done in a triplicate of 20 μl reaction using Phusion High‐Fidelity PCR MasterMix (Thermo Scientific, USA). The conditions were as follows: initial denaturation for 3 min at 98°C, 30 cycles of 98°C for 10 s, 52°C for 30 s, 72°C for 30 s, final elongation at 72°C for 5 min. The obtained triplicate reactions were pooled and amplicons were purified from the gel using the Wizzard SV Gel and PCR clean system (Promega) and quantified with Qubit dsDNA HS assay. Amplicons sequencing was performed on Illumina MiSeq (2 × 250bp) platform by Genomic Service of the Universitat Pompeu Fabra (Barcelona, Spain). Obtained *puf*M gene reads were processed in the same manner as 16S rRNA gene amplicons with different filterAndTrim function values (truncLen = c(130, 130), maxN = 0, maxEE = c(2,2), truncQ = 2, rm.phix = TRUE, compress = TRUE, multithread = FALSE). Final ASV table contained 468 pufM_ASVs and from 15,746 to 84,506 reads per sample (41,524 ± 12,440; Table S2). Taxonomic assignment was done in DADA2 (function: assignTaxonomy[]) using manually curated in‐house database. It contained 1475 unique *puf*M sequences, downloaded from the Fungene repository on May 16, 2019 (http://fungene.cme.msu.edu), from metagenomes from the Římov reservoir (Andrei et al., 2019; Mehrshad et al., 2018) and from the Genome Taxonomy database accessed on 16 September 2020 (Parks et al., 2018).

The sequences of 16S amplicons were deposited in the NCBI database under Biosamples SAMN26677261–SAMN26677286 and of *puf*M amplicons under Biosamples SAMN26677246–SAMN26677260 as a part of BioProject PRJNA816466.

### Phylogenetic analysis of *puf*M gene

The limitation of the taxonomic assignation of the *pufM* gene amplicons was evident from the initial classification using the default DADA2 algorithm, which failed to assign all Chloroflexota reads and 20% of Proteobacteria reads at class level, and 65% of Alphaproteobacteria reads at order level. Over 50% of reads remain unclassified at the order level after the DADA2 taxonomic assignment. Phylogenetic analysis reduced the contribution of these unclassified Alphaproteobacteria up to 95% in June. Moreover, even though only 5% of Gammaproteobacteria reads remained unclassified at order level, over a quarter of the Burkholderiales reads could not be assigned to the genus level. The *puf*M gene is more variable than 16S rRNA gene and the lack of reference sequences closely related to these unclassified pufM_ASVs in our database hampered a more precise taxonomic assignment.

To partially overcome this limitation and reveal the hidden diversity of freshwater AAP bacteria in the studied lake, we performed a phylogenetic analysis of these unclassified pufM_ASVs.

Amino acid sequences of pufM genes were obtained from the Genome Taxonomy database through AnnoTree tool (Mendler et al., 2019) on 13/09/2021. The sequences with percentage of identity ≤40% to the reference pufM sequence in GTDB were removed as they originated from the *pufL* gene. 14 *puf*M sequences from previous works (Fecskeová et al., 2019; Mujakić et al., 2021; Piwosz et al., 2020; Zeng et al., 2021), which are not shown in GTDB database but they represent a reference point in freshwater environments, were included in the analysis. The unclassified pufM_ASVs were translated to amino acids using the second forward open reading frame. Both the reference *puf*M sequences and the pufM_ASVs were aligned in Geneious v2019.2.3 using ClustalW v2.1 (Larkin et al., 2007). The phylogenetic analysis was done with IQTREE (Trifinopoulos et al., 2016). LG + F + I + G4 was selected as a best‐fit nucleotide substitution model by ModelFinder (Kalyaanamoorthy et al., 2017) according to Bayesian information criterion, and the Maximum Likelihood tree was calculated using 1000 ultrafast bootstrap replicates and default settings. The trees were prepared for the publication using iTOL (Letunic & Bork, 2021) and Inkscape v1.01.

### Statistical analysis

The statistical relationship between environmental data (Table T3), abundance of all bacteria and abundance of AAP bacteria was analysed by distance based linear models (DistML), distance based Redundancy Analysis (Anderson & Legendre, 1999; Legendre & Andersson, 1999) and non‐metric multi‐dimensional scaling (nMDS) in the PERMANOVA+ add‐on package of the PRIMER7 software (Anderson et al., 2008) (Primer Ltd., Lutton, UK). Environmental variables for each layer were averaged from the corresponding depths (0.5 and 2 m for the epilimnion and 5 to 9 m for the hypolimnion) and normalized. Correlations between different environmental variables were checked using Draftsman plots, and in the case of the strong correlation (absolute value of correlation coefficient >0.7) one of the variables was excluded from the analysis (Table T3). Abundance data for all and AAP bacteria (from microscopic counts), and the amplicon data (after removing singletons and doubletons) were log (*X* + 1) transformed. The best model was selected using a stepwise selection procedure based on statistical significance (9999 permutations) and the value of the Akaike's Information Criterion (AICc) (Anderson & Legendre, 1999; Legendre & Andersson, 1999).

## RESULTS AND DISCUSSION

### Environmental variables

Samples were collected from a freshwater lake Cep, from April to September. The environmental conditions were typical for oligo‐mesotrophic lakes (Figure S1) indicating that our results are representative for Northern hemisphere temperate lakes (Dodds & Whiles, 2020; Verpoorter et al., 2014). Thermal stratification of the water column was formed in May and present until the end of the sampling. Nevertheless, hypoxic or anoxic conditions were not observed in the hypolimnion (minimal oxygen concentration was 5.69 mg O_2_ L^−1^; Figure S1). Concentrations of nutrients and chlorophyll‐*a* (Chl‐*a*) were higher in the hypolimnion.

### Abundances of total and AAP bacteria

**FIGURE 1:**
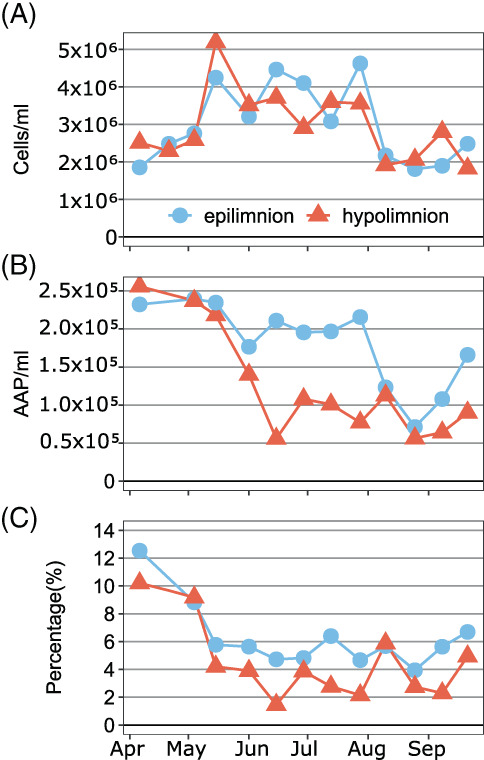
Weekly abundances of all prokaryotes (A), AAP bacteria (B) and relative abundance of AAP bacteria in the total bacterial community (C) in the epilimnion (blue‐circles and line) and the hypolimnion (red‐triangles and line) of Cep lake.

The abundance and dynamics of all bacteria were similar in the epi‐ and the hypolimnion, with maximum values in July for the epilimnion (4.62 × 10^6^ cells ml^−1^) and in May for the hypolimnion (5.19 × 10^6^ cells ml^−1^) (Figure 1A). The abundance of AAP bacteria ranged from 7.11 × 10^4^ to 2.39 × 10^5^ cells ml^−1^ in the epilimnion and from 5.57 × 10^4^ to 2.56 × 10^5^ cells ml^−1^ in the hypolimnion (Figure 1B), with the highest values in April and May in both layers. AAP bacterial abundance decreased in the hypolimnion in June, while in the epilimnion only in August (Figure 1B). The percentage contribution of AAP bacteria to the total bacterial abundance was generally higher in the epilimnion (3.93–12.53%) than in the hypolimnion (1.45–10.20%, Figure 1C). Such temporal patterns of AAP bacteria abundance between in the epi‐ and the hypolimnion are common in temperate freshwater lakes (Čuperová et al., 2013; Fauteux et al., 2015).

### Bacterial community composition

**FIGURE 2:**
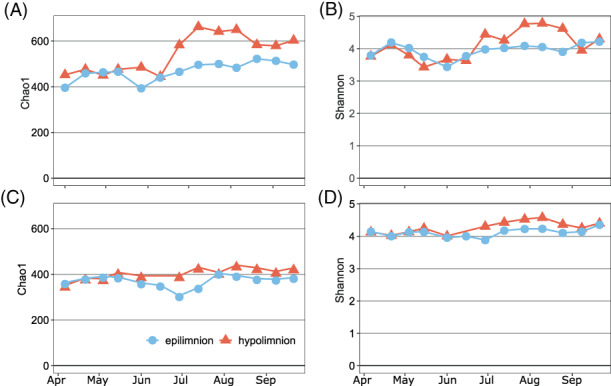
Weekly estimates of bacterial diversity in the epilimnion (blue‐circles and line) and the hypolimnion (red‐triangles and line) of Cep lake. (A) Chao1 index and (B) Shannon index estimated for total bacterial community based on 16S rRNA gene amplicon sequences; (C) Chao1 index and (D) Shannon index estimated for AAP bacterial community based on *puf*M gene amplicon sequences.

The bacterial alpha diversity was higher in the hypolimnion, especially in summer, based on 16S rRNA gene amplicons (16S_ASVs; Figure 2). The most abundant phyla were, in descending order, Actinobacteriota, Cyanobacteria, Proteobacteria, Bacteroidota, Verrucomicrobiota and Planctomycetota (Figure S2A). Non‐metric multidimensional scaling (nMDS) analysis showed that the largest differences in community composition between the two layers were during stratification period from June to September (Figure S2). Since the composition and temporal changes of bacterial community were typical for the oligo‐mesotrophic lakes (Cabello‐Yeves et al., 2017; Morrison et al., 2017), below we have just focused on phyla known to contain AAP bacteria: Gemmatimonadota, Chloroflexota and Proteobacteria.

**FIGURE 3:**
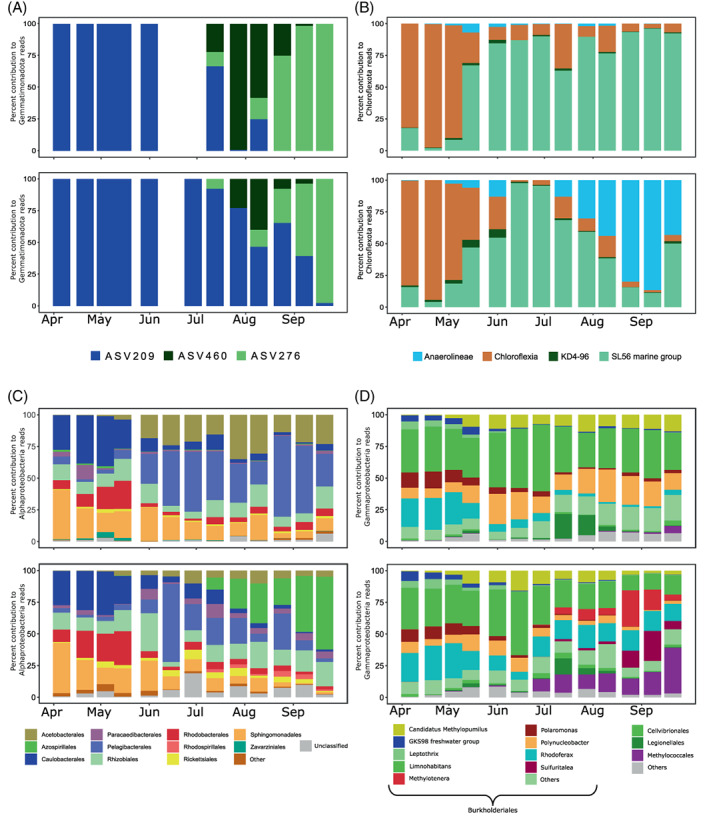
Bacterial community composition based on 16S rRNA gene amplicon sequencing in the epilimnion (upper plots in each panel) and the hypolimnion (bottom plots) in Cep lake. Only phyla and classes known to contain AAP species are shown. (A) Percent contribution of ASVs affiliated with Gemmatimonadota to the number of reads coming from Gemmatimonadota; (B) percent contribution of Chloroflexota of classes to the number of reads coming from Chloroflexota, (C) percent contribution of alphaproteobacterial orders to the number of reads coming from Alphaproteobacteria and (D) percent contribution of gammaproteobacterial orders to the number of reads coming from Gammaproteobacteria.

The relative abundance of Gemmatimonadota was <1%, which seems to be typical for freshwater lakes (Mujakić et al., 2021). Gemmatimonadota were represented by three 16S_ASVs (Figure 3A), classified as genus *Gemmatimonas* that is common in aquatic habitats (Gołębiewski et al., 2017; Mujakić et al., 2021). Their relative abundance varied substantially in both layers, with the changes in the epilimnion preceding those in the hypolimnion, suggesting a downward propagation of temporal changes (Cram et al., 2015).

Phylum Chloroflexota represented <4% of total 16S rRNA gene sequences (Figure S3A). Genera from class Chloroflexia known to contain *puf*M genes, such as *Roseiflexus*, *Kouleothrix* and UBA965 dominated during spring (Figure 3B). They were followed by the SL56 cluster (Ca. Limnocylindrus, Mehrshad et al., 2018) and they dominated in the epilimnion until the end of the sampling campaign. In the hypolimnion, a noticeable increase of the class Anaerolineae was observed in August and September (Figure 3B), when oxygen concentration was at its lowest. These bacteria are widely spread in different environments (Yamada & Sekiguchi, 2009) and were originally described to be anaerobic (Nakahara et al., 2019; Yamada et al., 2006) but recently an aerobic member containing type‐II reaction centre (RC) was assembled from a metagenome (Martinez et al., 2020).

Proteobacteria were the most abundant phylum with known AAP species (Figure S3A). Alphaproteobacteria and Gammaproteobacteria were the dominant classes, with a contribution to the Proteobacteria reads ranging from 23–49% and from 51–77%, respectively.

Temporal patterns of Alphaproteobacteria were quite distinct between the epi‐ and the hypolimnion (Figure 3C). Caulobacterales, Rhizobiales, Rhodobacterales and Sphingomonadales, orders known to contain AAP species (Imhoff et al., 2019; Kopejtka et al., 2017, 2021), dominated in spring, coinciding with the maxim values of AAP bacterial abundance. Pelagibacteriales dominated in the epilimnion and Azospirillales in the hypolimnion from June until the last sampling in September. They have already been reported as a part of microbial communities in a wide variety of freshwater environments (Galachyants et al., 2021; Tsementzi et al., 2019).

Gammaproteobacteria were dominated by the order Burkholderiales (Figure 3D), specifically by genera such as *Limnohabitans*, *Rhodoferax* and *Polynucleobacter*, important members of microbial food webs also known to contain AAP species (Hahn et al., 2012; Jezberová et al., 2017; Kasalický et al., 2018; Salka et al., 2011; Šimek et al., 2005). From the onset of stratification, the Gammaproteobacteria community started to develop differently in the depth layers. The relative abundance of Ca. Methylopumilus and *Polynucleobacter* increased in the epilimnion, while *Methylotenera*, *Sulfuritalea* and order Methylococcales increased in the hypolimnion.

### AAP bacteria community composition

The AAP community composition was assessed with amplicon sequencing of the *puf*M gene, which is a routinely used marker for anoxygenic phototrophs containing type‐II RC (Koblížek, 2015). The number of *puf*M ASVs (pufM_ASVs) estimated using the Chao1 index and the Shannon diversity index were higher in the hypolimnion than in the epilimnion (Figure 2C, D). Higher bacterial diversity in the hypolimnion seems to be a common feature of freshwater lakes (Shade et al., 2012), and here we demonstrated that this may be also a trend for AAP bacteria, at least in shallow lakes that are oxygenated and illuminated to the bottom. Moreover, AAP community started to diverge between the epi‐ and the hypolimnion in summer (Figure S2B), as observed also for all bacteria. Nevertheless, the difference in alpha and beta diversity between two layers was less conspicuous for AAP bacteria than for the total bacterial community (Figure 2 and Figure S1).

Gemmatimonadota, Chloroflexota and Proteobacteria were the only phyla detected in our samples, while none of the phototrophic members of Eremiobacteriota and Myxococcota were present in the *puf*M gene libraries (Figure S3B).

**FIGURE 4:**
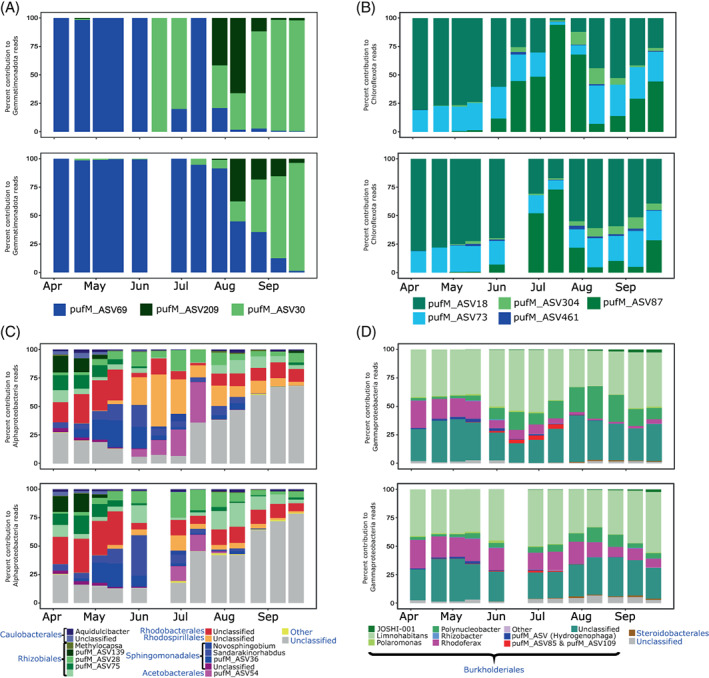
AAP bacteria community composition based on *puf*M gene amplicon sequencing in the epilimnion (upper plots in each panel) and the hypolimnion (bottom plots) in Cep lake. (A) Percent contribution of ASVs affiliated with Gemmatimonadota to the number of reads coming from Gemmatimonadota; (B) percent contribution of ASVs affiliated with Chloroflexota to the number of reads coming from Chloroflexota, (C) percent contribution of alphaproteobacterial genera and ASVs to the number of reads coming from Alphaproteobacteria and (D) percent contribution of gammaproteobacterial genera and ASVs to the number of reads coming from Gammaproteobacteria.

The contribution of Gemmatimonadota to the AAP bacteria community was in general below 2%, except in September, when it increased to 9% (Figure S3B). Gemmatimonadota community consisted of three pufM_ASVs (Figure 4A). pufM_ASV69, that clustered in the phylogenetic tree together with the environmental cluster Pg2 (Mujakić et al., 2021) (Figure S4), dominated in spring in the epilimnion and in spring and summer in the hypolimnion (Figure 4A). It was almost replaced by pufM_ASV30 and pufM_ASV209 (Figure S4) that, despite being closely related, showed a distinct seasonal pattern (Figure 4A). Such temporal separation of closely related phylotypes may have resulted from the differences in their physiology, as observed for *G. phototrophica* and *G. groenlandica* (Zeng et al., 2021). Interestingly, the dynamics of Gemmatimonadota by pufM_ASVs resembles that of 16S_ASVs (Figure 3A and Figure 4A). This suggests that pufM_ASV209, pufM_ASV30 and pufM_ASV69 from *puf*M amplicons might correspond to 16S_ASV460, 16S_ASV276 and 16S_ASV209 from 16S rRNA gene amplicons, respectively.

Chloroflexota contributed up to 5% to the *puf*M gene sequences and were more abundant in April and July–August (Figure S3B). They consisted of 5 pufM_ASVs from an uncultured freshwater clade of the Roseiflexaceae family (Mehrshad et al., 2018) that differed only by 1 or 2 amino acids (Figure S5). Despite this high similarity, these Chlorofleoxta ASVs showed distinct dynamics (Figure 4B). Interestingly, the most abundant pufM_ASV18 and pufM_ASV87 were shown to be highly active in August 2016 in the same lake, based on the comparison of DNA and RNA amplicon libraries (Fecskeová et al., 2019). Our results indicate that these Chloroflexota are core members of freshwater AAP bacteria communities over the whole sampling season.

Proteobacteria represented over 90% of all *puf*M reads (Figure S3B). This comprised between 2% and 14% of Alphaproteobacteria, while Gammaproteobacteria were always more than 50%, with a maximum >90% in April (Figure S2B).

Alphaproteobacteria community was similar in both layers in spring, being dominated by Rhodobacterales, Rhizobiales and Caulobacterales (Figure 4C), as observed also for 16S amplicons (Figure 4D). From the onset stratification, Rhodospirillales increased their contribution in the epilimnion, while Sphingomonadales and Rhizobiales showed their maximum contribution in the hypolimnion. Interestingly, the contribution of unclassified reads increased from July, and they reached up to 75% by late September (Figure 4C).

Phototrophic Gammaproteobacteria were more stable compared with other phototrophic phyla, and there was little difference between the epi‐ and the hypolimnion (Figure 4D). Burkholderiales made up >90% of reads in all samples. *Limnohabitans*, *Polynucleobacter* and *Rhodoferax* were the dominant genera, but 20–25% of the Burkholderiales remained unclassified at genus level. *Limnohabitans* and *Rhodoferax* showed the highest relative abundances in April until June, whereas *Polynucleobacter* showed this in August and September, reaching almost 30% of the Gammaproteobacteria reads in the epilimnion. Phototrophic activity of Burkholderiales may have been relatively low, as they were substantially underrepresented in the active AAP community investigated in August 2016 in the same lake (Fecskeová et al., 2019).

Most of the groups recovered in our time series were also reported in snapshots studies of freshwater lakes (Cepáková et al., 2016; Čuperová et al., 2013; Fecskeová et al., 2019). Here, we show that the composition of AAP community substantially varies in both epi‐ and hypolimnion, with many groups showing transient occurrence (Figure 4). This clearly indicated that the snapshot studies cannot sufficiently describe their diversity and the season of sampling affects the results. As confirmation, a substantial proportion of the reads and pufM_ASVs in our study remained unassigned at genus or order level (Figure 4). This was not just the case for the understudied phyla, such as Chloroflexota and Gemmatimonadota, but also for Proteobacteria (Figure 4C, D), for which diversity in freshwater environments is well described (Ferrera et al., 2017). Furthermore, some pufM_ASVs could not be classified even to the class level. For instance, a group of 20 pufM_ASVs formed a branch between Rhodobacterales (Alphaproteobacteria) and Ectothiorhodospirales (Gammaproteobacteria) (Figure S6). The lack of reference sequences related to the unclassified pufM_ASVs hampered the taxonomic assignation of many of them. These results indicate the high potential of undescribed diversity of AAP bacteria in freshwaters that can be investigated for instance using metagenomics (Mehrshad et al., 2018; Mujakić et al., 2021; Ward et al., 2019).

### Relationship with environmental variables

Temporal dynamics of AAP bacteria abundance has been shown to respond to changes in the environmental conditions (Kolářová et al., 2019; Lew et al., 2015; Mašín et al., 2008). However, how they influence the AAP community composition remains mostly unknown. Such information is important as, for example, high photoheterotrophic activity by AAP bacteria was linked to elevated relative abundance of few alphaproteobacterial orders: Caulobacteriales and Sphingomonadales (Piwosz et al., 2022). Thus, we conducted statistical analysis to reveal environmental factors that may have influenced the dynamics of AAP communities.

**FIGURE 5:**
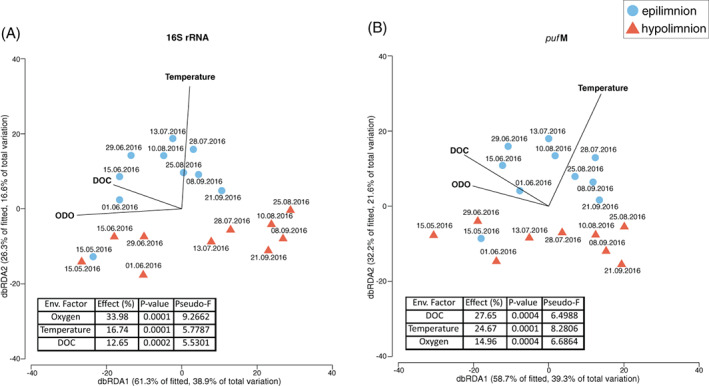
Distance‐based redundancy analysis biplots relating the observed variability in the composition of communities for (A) all bacteria and for (B) AAP bacteria to the explanatory variables (black lines) in the epilimnion (blue‐circles) and hypolimnion (red‐triangles). Tables embedded in the graphs show the percentage of variability explained (effect [%]) by individual environmental variables (Env. Factor) and their statistical significance (*p* value and pseudo‐F). DOC, dissolved organic carbon; ODO, optical dissolved oxygen.

Distance‐based linear models (DistLM) and distance‐based redundancy analysis (dbRDA) (Anderson & Legendre, 1999; Legendre & Andersson, 1999) showed that the environmental factors that best explained the variability of the total bacterial community were oxygen, temperature and dissolved organic carbon (DOC) (Figure 5). The variability in the AAP community composition was explained by the same environmental variables but the explanatory power of particular variables differed. Namely, the best explanatory variable was DOC, followed by temperature, whereas oxygen, which explained most of the variability in the total bacterial community was less important for AAP bacteria. These environmental factors have been shown to be determinants in general for shaping bacterial and AAP communities (Ferrera et al., 2017; Mašín et al., 2008; Niño‐García et al., 2016). Nevertheless, the observation that this functional group correlates differently with those variables than total bacteria indicates that their response to changing environmental conditions may differ as well.

## SUMMARY AND CONCLUSIONS

In this report, we investigated temporal changes in the diversity of bacteria in a freshwater lake, with the focus on AAP bacteria. Our results showed that the AAP community followed different temporal patterns in the epilimnion and the hypolimnion when the lake was stratified. The high number of unclassified reads in the *puf*M libraries indicates that there is still hidden diversity of AAP bacteria within the known bacterial phyla. This was the case not only for less known Gemmatimonadota and Chloroflexota but, surprisingly, also for Alphaproteobacteria, the class to which the first described AAP species belongs (Harashima et al., 1978). Progress in linking this unexpected *puf*M diversity with taxonomic affiliation may be expected with increasing use of metagenomics, targeted single‐cell sequencing and culturing of AAP bacteria (Parks et al., 2017; Woyke et al., 2017).

Environmental conditions shape bacterial communities, resulting in temporal changes in their diversity and composition, driven by adaptation that causes physiologic differences between distinct phylotypes. In contrast to our expectations, the same set of environmental factors explained variability of all and AAP communities but they differed in their importance for total versus functional group. This indicates that AAP bacteria may respond differently to the changing environment, for example to temperature rise and deoxygenation of lakes due to global warming. More information on metabolic capacity of this photoheterotrophic bacteria is needed to fully understand their dynamics.

## AUTHOR CONTRIBUTIONS

Cristian Villena‐Alemany: analysis of the sequencing data, prepared figures and writing the manuscript; Izabela Mujakić: statistical analysis, prepared the amplicons, prepared a figure and commenting on the manuscript; Petr Porcal: measurement of environmental chemicals and commenting on the manuscript; Michal Koblížek: editing and commenting on the manuscript; Kasia Piwosz: participated in sampling, extracted DNA, supervised analysis of data, editing and commenting on the manuscript.

## Supporting information


**FIGURE S1.** Weekly values of environmental variables in the epilimnion (blue‐circles and line) and the hypolimnion (red‐triangles and line) of Cep lake. (A) temperature; (B) optical dissolved oxygen; (C) dissolved organic carbon; (D) soluble reactive phosphorous; (E) dissolved nitrogen and (F) chlorophyll‐*a*.
**FIGURE S2.** Nonmetric multidimensional scaling plots of (A) all bacteria and (B) AAP bacteria based on Bray‐Curtis distances.
**FIGURE S3.** Community composition at the phylum level of (A) total bacteria (based on 16S rRNA gene amplicon sequencing) and (B) AAP bacteria (based on *puf*M gene amplicon sequencing) for the epilimnion (top panel) and the hypolimnion (bottom panel).
**FIGURE S4.** Details of Gemmatimonadota sequences distribution in the PufM protein Maximum Likelihood phylogenetic tree computed using LG + F + I + G4 substitution model. The analysis involved pufM amino acid sequences of the reference sequences from Proteobacteria, Chloroflexota, Gemmatimonadota and Eremiobacteriota and the targeted unclassified ASVs.
**FIGURE S5.** Details of Chloroflexota sequences distribution in the PufM protein Maximum Likelihood phylogenetic tree computed using LG + F + I + G4 substitution model. The analysis involved pufM amino acid sequences of the reference sequences from Proteobacteria, Chloroflexota, Gemmatimonadota and Eremiobacteriota and the targeted unclassified ASVs.
**FIGURE S6.** Details of Proteobacteria sequences distribution in the PufM protein Maximum Likelihood phylogenetic tree computed using the LG + F + I + G4 substitution model. The analysis involved pufM amino acid sequences of the reference sequences from Proteobacteria, Chloroflexota, Gemmatimonadota and Eremiobacteriota and the targeted unclassified ASVs.

**TABLE S1.** The number of reads per ASV in each sample (ASV_Table) and taxonomy assignment of each ASV (Taxa_Table) for 16 S rRNA gene amplicons.

**TABLE S2.** The number of reads per ASV in each sample (ASV_Table) and taxonomy assignment of each ASV (Taxa_Table) for *puf*M gene amplicons.

**TABLE T3.** Environmental data taken from the epilimnion and hypolimnion of Cep lake during 2016. Second sheet shows values of Pearson correlation coefficient for environmental variables for both 16 S rRNA and *puf*M genes. In case of a strong correlation (absolute value of Pearson correlation coefficient >0.7, marked with red), the variable was excluded from further DistLM analysis. Third sheet shows DistLM model with values (pseudo‐F, p) for statistically significant environmental variables.
