# Supplementary material for: Diversity dynamics of aerobic anoxygenic phototrophic bacteria in a freshwater lake
Source: Environ Microbiol Rep. 2022 Dec 12;15(1):60–71. doi: 10.1111/1758-2229.13131 (PMC10103773; doi:10.1111/1758-2229.13131)
Supplement: Supplementary file 1 — FIGURE S1. Weekly values of environmental variables in the epilimnion (blue‐circles and line) and the hypolimnion (red‐triangles and line) of Cep lake. (A) temperature; (B) optical dissolved oxygen; (C) dissolved organic carbon; (D) soluble reactive phosphorous; (E) dissolved nitrogen and (F) chlorophyll‐a. FIGURE S2. Nonmetric multidimensional scaling plots of (A) all bacteria and (B) AAP bacteria based on Bray‐Curtis distances. FIGURE S3. Community composition at the phylum level of (A) total bacteria (based on 16S rRNA gene amplicon sequencing) and (B) AAP bacteria (based on pufM gene amplicon sequencing) for the epilimnion (top panel) and the hypolimnion (bottom panel). FIGURE S4. Details of Gemmatimonadota sequences distribution in the PufM protein Maximum Likelihood phylogenetic tree computed using LG + F + I + G4 substitution model. The analysis involved pufM amino acid sequences of the reference sequences from Proteobacteria, Chloroflexota, Gemmatimonadota and Eremiobacteriota and the targeted unclassified ASVs. FIGURE S5. Details of Chloroflexota sequences distribution in the PufM protein Maximum Likelihood phylogenetic tree computed using LG + F + I + G4 substitution model. The analysis involved pufM amino acid sequences of the reference sequences from Proteobacteria, Chloroflexota, Gemmatimonadota and Eremiobacteriota and the targeted unclassified ASVs. FIGURE S6. Details of Proteobacteria sequences distribution in the PufM protein Maximum Likelihood phylogenetic tree computed using the LG + F + I + G4 substitution model. The analysis involved pufM amino acid sequences of the reference sequences from Proteobacteria, Chloroflexota, Gemmatimonadota and Eremiobacteriota and the targeted unclassified ASVs. [file EMI4-15-60-s001.pdf]

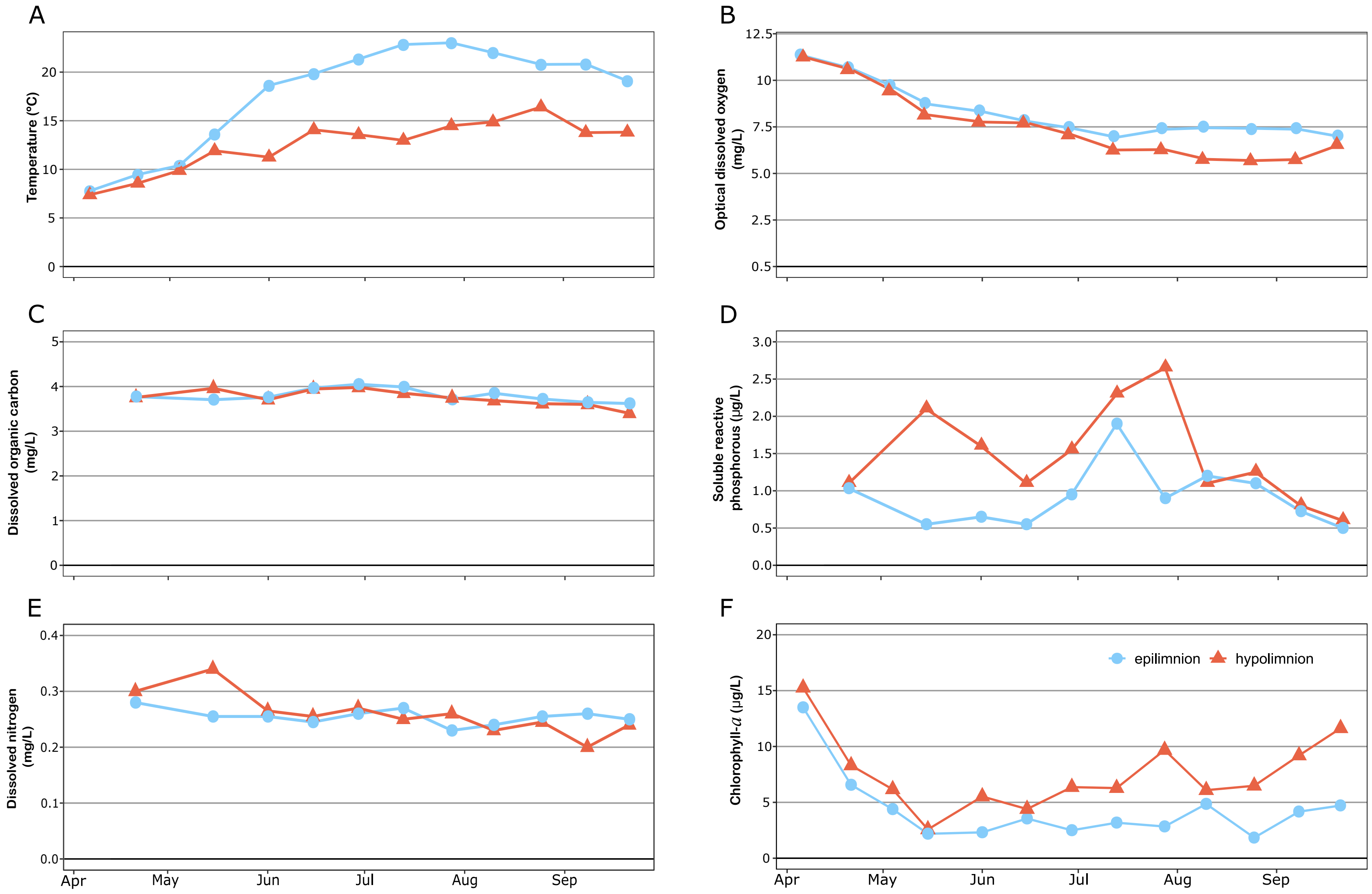

Supplementary Figure S1. Weekly values of environmental variables in epilimnion (blue-circles and line) and hypolimnion (red - triangles and line) of Cep lake. (A) temperature; (B) optical dissolved oxygen; (C) dissolved organic carbon; (D) soluble reactive phosphorous; (E) dissolved nitrogen; and (F) chlorophyll-a.

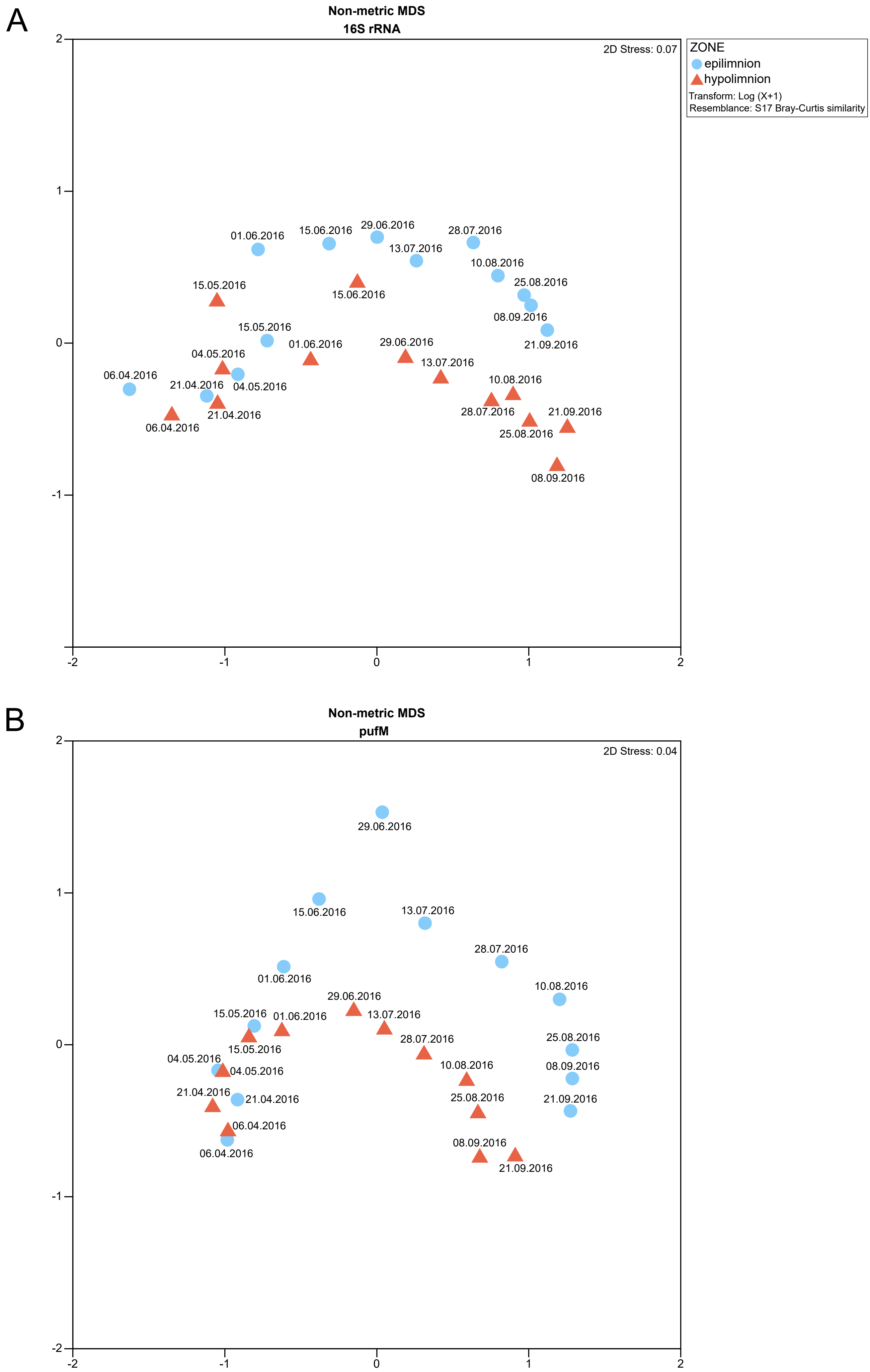

Supplementary Figure S2. Nonmetric multidimensional scaling plots of (A) all bacteria and (B) AAP bacteria based on Bray-Curtis distances.

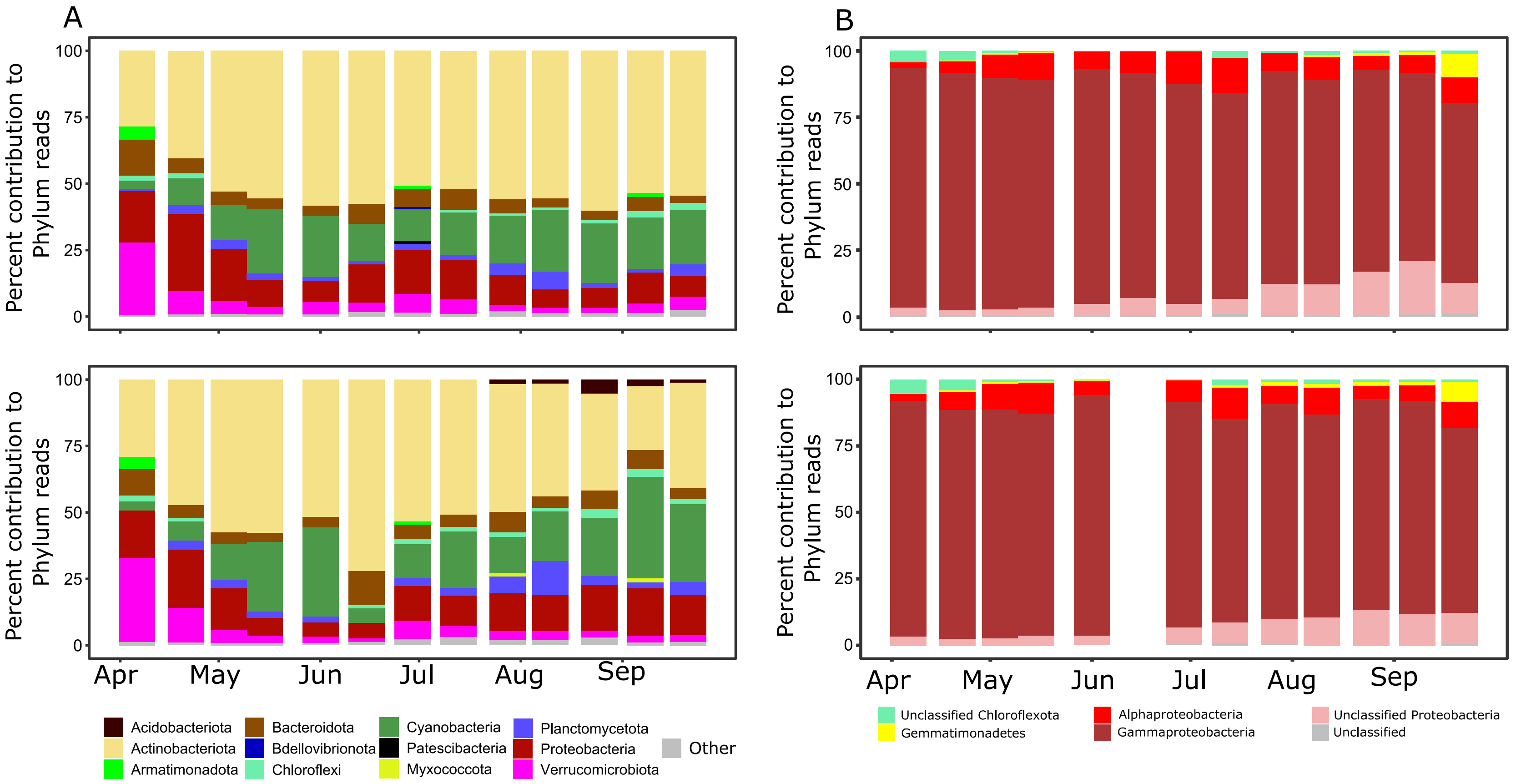

Supplementary Figure S3. Community contribution at phylum level of (A) total bacteria community and (B) AAP bacteria community for epilimnion (top) and hypolimnion (bottom).

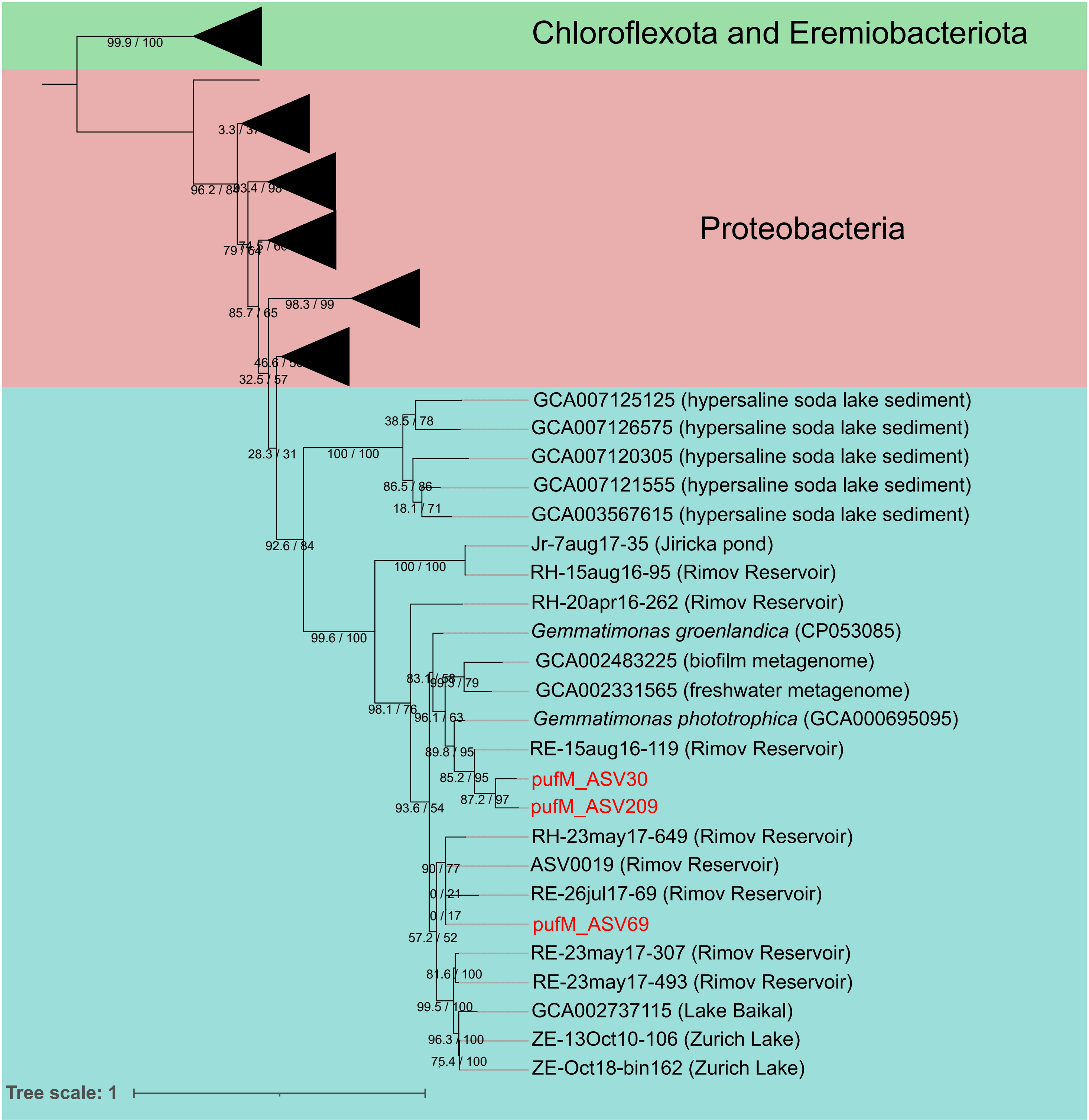

Supplementary Figure S4. Phylogenetic tree showing in detail Gemmatimonadota phylum sequences and unclassified ASVs that clustered inside.

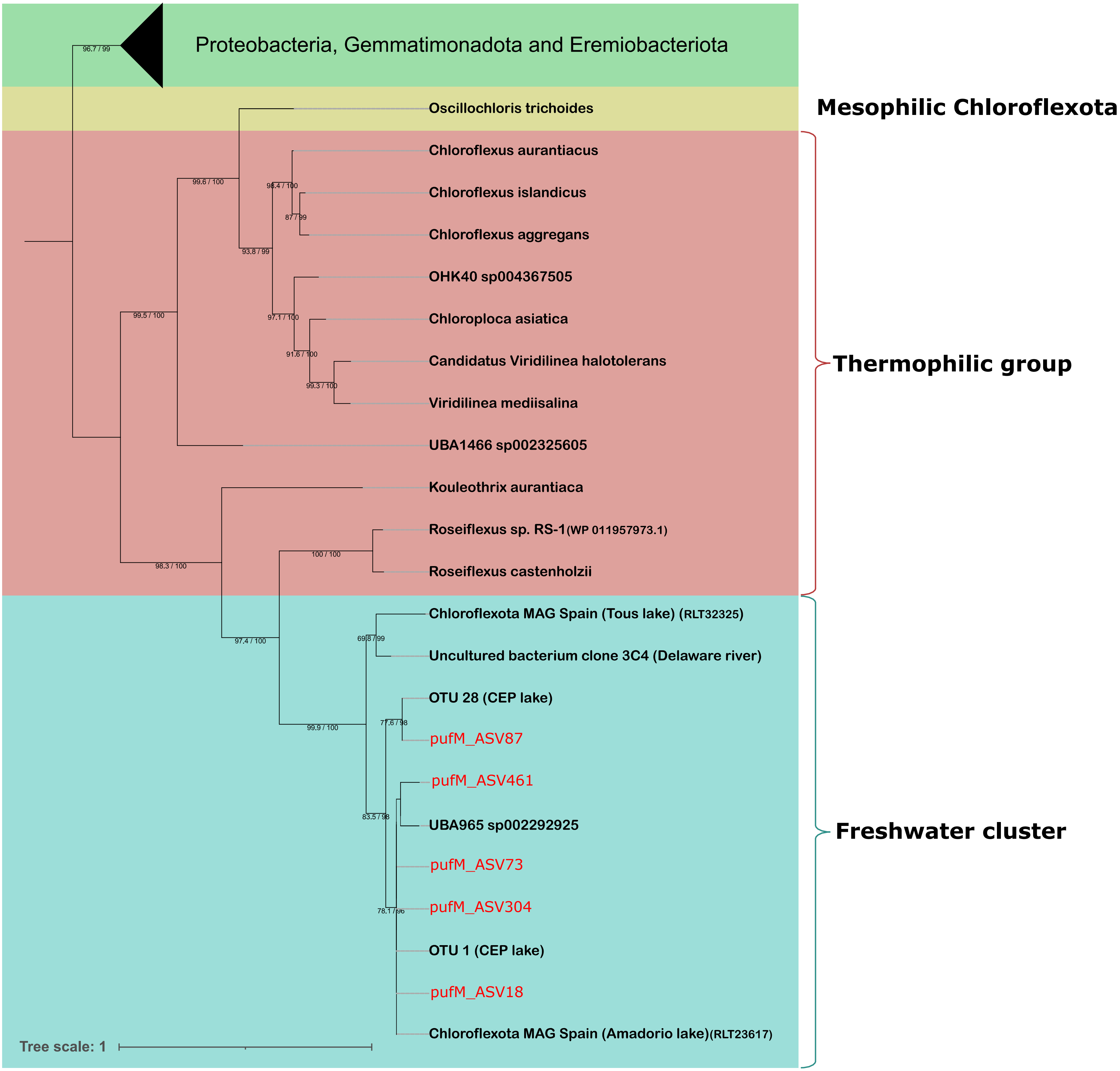

Supplementary Figure S5. Details of Chloroflexota sequences distribution in the pufM protein Maximum Likelihood phylogenetic tree computed using LG+F+I+G4 substitution model. The analysis involved pufM amino acid sequences of the reference sequences from Proteobacteria, Chloroflexota, Gemmatimonadota and Eremiobacteriota and the targeted unclassified ASVs.

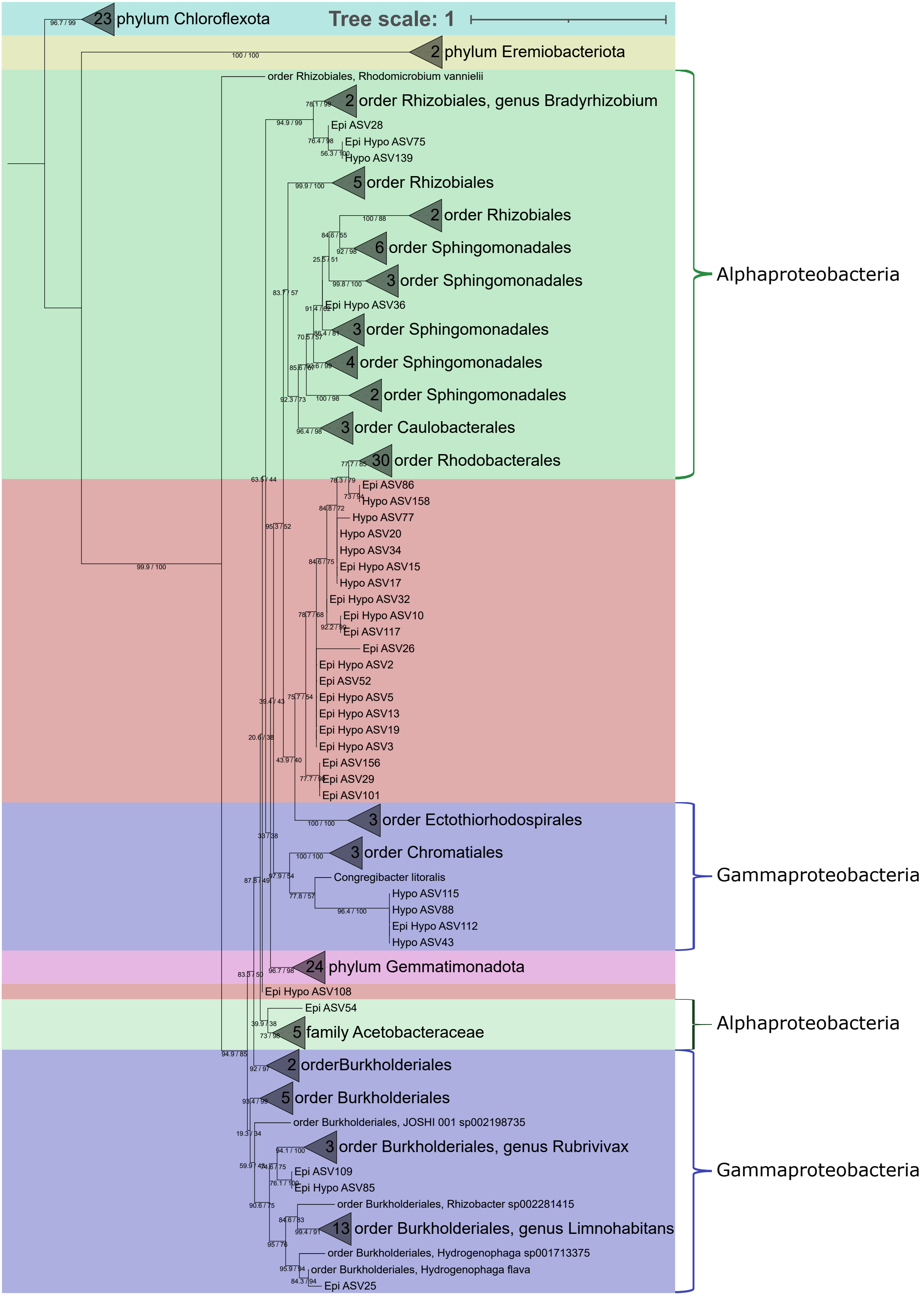

Supplementary Figure S6. Phylogenetic tree showing in detail Proteobacteria phylum sequences and unclassified ASVs that clustered inside.
